# Who consults chiropractors in Victoria, Australia?: Reasons for attending, general health and lifestyle habits of chiropractic patients

**DOI:** 10.1186/s12998-016-0110-2

**Published:** 2016-09-01

**Authors:** Melanie J. Charity, Helena C. Britt, Bruce F. Walker, Jane M. Gunn, Kirsty Forsdike-Young, Barbara I. Polus, Simon D. French

**Affiliations:** 1 Department of General Practice, University of Melbourne, Melbourne, Australia; 2 Institute of Sport Exercise and Active Living, Victoria University, Melbourne, Australia; 3 School of Health Sciences, Federation University, Ballarat, Australia; 4 Family Medicine Research Centre, Sydney School of Public Health, University of Sydney, Sydney, Australia; 5 School of Health Professions, Murdoch University, Murdoch, Australia; 6 Research and Innovation, RMIT University, Melbourne, Australia; 7 School of Rehabilitation Therapy, Queen’s University, Louise D. Acton Building, 31 George St, Kingston, ON K7L 3N6 Canada

**Keywords:** Practice-based research, Cross-sectional study, Chiropractic, Public health

## Abstract

**Background:**

COAST (Chiropractic Observational and Analysis STudy) reported the clinical practices of chiropractors. The aims of this study were to: 1) describe the chiropractic patient demographic and health characteristics; 2) describe patient-stated reasons for visiting a chiropractor; 3) describe chiropractic patient lifestyle characteristics; 4) compare, where possible, chiropractic patient characteristics to the general Australian population.

**Methods:**

Fifty-two chiropractors in Victoria, Australia, provided information for up to 100 consecutive encounters. If patients attended more than once during the 100 encounters, only data from their first encounter were included in this study. Where possible patient characteristics were compared with the general Australian population.

**Results:**

Data were collected from December 2010 to September 2012. Data were provided for 4464 encounters, representing 3287 unique individuals. The majority of chiropractic encounters were for musculoskeletal conditions or for wellness/maintenance. The majority of patient comorbidities were musculoskeletal, circulatory or endocrine/metabolic in nature. Eight hundred chiropractic patients (57 %, 95 % CI: 53–61) described their self-reported health as excellent or very good and 138 patients (10 %, 95 % CI: 8–12) as fair or poor. Seventy-one percent of adult male patients (18 years and older), and 53 % of adult female patients, were overweight or obese. Fourteen percent (*n* = 188, 95 % CI: 12–16) were current smokers and 27 % (*n* = 359, 95 % CI: 24–31) did not meet Australian alcohol consumption guidelines. Less than half of the chiropractic patients participated in vigorous exercise at least twice per week. Approximately 20 % ate one serving of vegetables or less each day, and approximately 50 % ate one serve of fruit or less each day. Compared to the general Australian population, chiropractic patients were less likely to smoke, less likely to be obese and more likely to describe their health in positive terms. However, many patients were less likely to meet alcohol consumption guidelines, drinking more than is recommended.

**Conclusions:**

In general, chiropractic patients had more positive health and lifestyle characteristics than the Australian population. However, there were a significant proportion of chiropractic patients who did not meet guideline recommendations about lifestyle habits and there is an opportunity for chiropractors to reinforce public health messages with their patients.

## Background

There are more than 5000 chiropractors practising in Australia [1] and approximately 16 % of Australians consult a chiropractor each year [2, 3]. However, little is known about the patients who seek chiropractic care in Australia and why they seek that care.

Previous studies of patient profiles of chiropractic patients have mostly been based in North America and Europe [4–7], or have focussed on specific patient groups rather than general patient characteristics [8–10]. Little is known about the health characteristics of chiropractic patients in Australia, and how their health compares to that of the general Australian population. It is important to have up to date information about the health characteristics of the patients who consult with chiropractors to guide the profession’s educators, researchers and policy makers about where to focus their activities.

COAST (Chiropractic Observational and Analysis STudy) aimed to report information about the Australian chiropractic profession [11]. A previous COAST paper reported the demographic details of participating chiropractors, the reasons people consulted chiropractors, and the treatment chiropractors provided. In this paper, we report COAST patient participant data with the following aims: 1) to describe the chiropractic patient demographic and health characteristics; 2) to describe patient-stated reasons for visiting a chiropractor; 3) to describe chiropractic patient lifestyle characteristics; and, 4) to compare, where possible, chiropractic patient characteristics to the general Australian population.

## Methods

Data for this study were collected as part of COAST, a cross-sectional observational study that described chiropractic practice in Victoria, Australia. The full methods of the study are published elsewhere [11]. In brief, a random sample of 180 chiropractors from the list of the 1298 registered chiropractors in Victoria, Australia, were approached to participate [12]. Chiropractors were included if they currently practiced in Victoria, but were excluded if they were in locum practice. Chiropractors were asked to record consecutive patient encounters until 100 encounters were recorded, or when 4 weeks of recording had elapsed. For this study, repeat patient visits within a chiropractor’s set of 100 encounters were identified by matching date of birth, gender and postcode. Data were collected from December 2010 to September 2012.

Participating chiropractors recorded anonymous patient encounter data on structured paper encounter recording forms using both check boxes and free text. Each chiropractor received a telephone call from a research assistant to provide training in completing the encounter forms, and was given detailed printed instructions. Data were classified and entered in a database by a coder according to the International Classification of Primary Care, Version 2 (ICPC-2) through the Australian ‘PLUS’ general practice terminology (ICPC-2 PLUS) [13, 14], with additional terms relevant to chiropractic practice generated throughout the study [15].

### Measures

#### Patient demographic and health characteristics

Participating chiropractors recorded each patient’s stated reasons for encounter (RFE), comorbidities, date of birth, gender, height and weight, postcode of residence, occupation and health insurance status. Body Mass Index (BMI) was calculated for all those with self-reported height and weight data.

We determined the most common reasons for a patient to visit a chiropractor, along with the most common comorbidity present at that visit. For this study, a comorbidity was defined as any existing health condition that was present at the time of the visit, but was not the RFE. Up to three RFEs and up to three comorbidities could be recorded at each encounter.

Deciles from the Australian Socio-economic Indexes for Areas (SEIFA) for Relative Socio-economic Advantage and Disadvantage (RSAD) were assigned where a patient’s residential postcode was provided. RSAD scores take into account a range of individual factors including household income and level of education which are then summarised for the geographic area. Higher SEIFA scores are associated with relative advantage and lower scores with relative disadvantage. Deciles are then assigned to an area from the lowest 10 % through to the highest 10 % by SEIFA score, resulting in 10 categories with equal numbers [16].

#### Patient lifestyle characteristics

The encounter recording forms included items about each patient’s lifestyle and general health. These items were located along the bottom part of the form and a different set of questions alternated sequentially between two formats in the total 100 forms provided to the chiropractor. One encounter form (the odd numbered encounter forms) included items about general health, quality of life and the extent that pain affected activities. The second format (on the even numbered encounter forms) included questions about fruit and vegetable intake, smoking, the number of standard drinks consumed in the last 7 days, and the amount of vigorous and non-vigorous exercise undertaken. Vigorous exercise included activities such as netball, squash, jogging, aerobics, and vigorous swimming. Less vigorous exercise included activities such as walking, gardening, swimming and lawn bowls. Due to the alternating nature of the encounter forms, each set of questions were completed for approximately half of all included patients.

Alcohol consumption was measured by an item that requested adult participant’s (18 years and older) alcohol intake over the last 7 days, with the number of drinks on separate days recorded. Participants were defined as meeting the alcohol consumption guidelines if they drank “no more than two standard drinks on any day” [17]. This is the rate of alcohol consumption that “reduces the lifetime risk of harm from alcohol-related disease or injury” as per guidelines from the Australian National Health and Medical Research Council (NHMRC) [17].

Where possible, we determined the differences and similarities between lifestyle characteristics of included patients and summary results of the 2011-12 Australian Health Survey published by the Australian Bureau of Statistics. The Australian Health Survey results were used for comparison as the survey is considered to be representative of the Australian population. In addition, we used the questions from the survey to develop the COAST patient lifestyle questions, which were then modified to function within the encounter form format. The Australian Health Survey is a combination of three Australian surveys: National Health Survey (NHS); the National Nutritional and Physical Activity Survey (NNPAS); and the National Health Measures Survey (NHMS) [18–20]. A core group of 33,500 people answered questions common to the NHS and NNPAS, including questions on health status, Body Mass Index, smoking, and fruit and vegetable consumption. Physical activity and alcohol consumption were addressed in the NHS (*n* = 20,500) [18–20]. Summary results of the Australian Health Survey characteristics were reported for participants 18 years of age and older, except general health, which was reported for participants aged 15 or more. COAST results were age-matched to these parameters.

### Statistical analysis

Descriptive statistics were used to summarise patient characteristics. Analyses adjusted for the clustered nature of the design (with multiple observations collected per chiropractor) and 95 % confidence intervals (CI) were calculated for all relevant estimates. Where repeat visits occurred within chiropractors’ 100 recorded encounters, information about the patient from only the first encounter form was used in this current analysis.

In previously published COAST results, the encounter was the unit of analysis [12]. However, for the analysis reported in this paper, repeat patient encounters during the recording period were removed before analysis, making the patient the unit of analysis. Lifestyle variables were reported as a percentage of all included patients. RFEs were organised into chiropractic-specific groups (further details reported elsewhere [15]) and were reported as percentage of patients with at least one RFE in a selected group. Where a patient had more than one comorbidity classified in the same ICPC chapter, only one was counted. Results are reported as a percentage of patients with at least one comorbidity in a selected chapter.

COAST patient participants were compared to those in the Australian Health Survey for the following characteristics: BMI, smoking status, self-reported health and alcohol consumption. As comparisons were made between case data and summary data, one sample t-tests were used to investigate differences between COAST patient participants and the general population (Australian Health Survey) for dichotomous variables (gender & alcohol consumption) and *χ*^2^ goodness-of-fit tests were used to investigate variables with multiple levels (BMI, smoking status, health rating). Due to an over representation of COAST participants with higher SEIFA RSAD scores, cases were weighted inversely according to SEIFA value. Analyses were conducted using Stata version 13 and SPSS Version 22 [21, 22].

The project was approved by the University of Melbourne Human Research Ethics Committee (HREC 0931651: Chiropractic in Australia), and all participants provided informed consent.

## Results

Seventy two chiropractors agreed to participate (46 % response rate of eligible chiropractors approached). During the study, 20 (28 %) of these chiropractors withdrew and did not provide any data. Fifty two chiropractors (72 % of those enrolled) completed the study, providing information for 4464 chiropractor-patient encounters. Of these, 1123 (25 %) encounters were identified as repeat patient encounters during the recording period and were removed from further analyses, leaving 3287 unique patients. A further 54 encounters did not have a date of birth recorded and were also removed. The odd numbered lifestyle questions were completed for 1594 patients, with 1403 of these aged 18 years and over. The even numbered lifestyle questions were completed for 1563 patients, with 1388 aged 18 and over, and 1442 aged 15 or more. Not all chiropractors provided 100 encounters: 33 out of 52 provided at least 100, 13/52 provided between 50 and 100, and 6/52 provided less than 50 encounters.

According to available demographic characteristics, participating chiropractors appeared to be representative of the broader Australian chiropractic population. Compared with all chiropractors in Victoria, COAST chiropractors had been practising a similar time (17 years), and a similar proportion worked in an urban location (35/52 [67 %] compared with 626/894 [70 %]). However, a smaller proportion of COAST participants were female (14/52 [27 %] compared with 399/1050 [38 %]), but this difference was not statistically significant. Compared with all Australian chiropractors, COAST chiropractors were of similar age but there were fewer female chiropractors than nationally (14/52 [27 %] compared with 1679/4664 [36 %]); this difference was not statistically significant.

Demographic details of the patients who sought chiropractic care are shown in Table 1. Over half of the patients were female (56 %), and the majority of patients seen were between the ages of 25 to 64 (71 %). Ten percent of patients were less than 15 years old; of these, 5 % were less than 5 years old. Those aged 65 years and older made up 12 % of patients. The majority of patients were either employed or students (79 %), and less than half (47 %) paid for their consultation using private health insurance. Of the chiropractic patients with valid postcodes, 50 % were in the three least disadvantaged SEIFA deciles.

**Table 1:** Demographic details of COAST patients

	Number	% of patients^a^ (*n* = 3287)	95 % confidence interval
Gender			
[missing]	[77]		
Female	1789	56	(53,58)
Male	1421	44	(42,47)
Age			
[missing]	[27]		
0–4	156	5	(2,10)
5–14	154	5	(4,6)
15–24	243	7	(6,9)
25–44	1161	36	(32,39)
45–64	1142	35	(32,38)
65–74	270	8	(7,10)
75+	134	4	(3,6)
Employment			
[missing]	[350]		
Employed/student	2307	79	(75,82)
Not working	16	1	(3,10)
Other^b^	614	21	(18,24)
Private health insurance			
[missing]	[128]		
Yes	1497	47	(40,56)
No	1662	53	(45,61)
SEIFA RSAD			
[missing]	[60]		
1 (most disadvantaged)^c^	64	2	(1,4)
2	183	6	(2,15)
3	261	8	(4,15)
4	249	8	(4,15)
5	261	8	(5,13)
6	293	9	(6,14)
7	303	9	(7,13)
8	345	11	(7,16)
9	694	22	(17,28)
10 (least disadvantaged)^c^	574	18	(12,26)

^a^Missing data not used in percentage calculations

^b^Includes home duties & retirement

^c^Relative to other deciles

The distribution of patient RFE groups is shown in Table 2. Health maintenance/preventative care (including maintenance care and check-up) was the most common RFE group, recorded for 39 % of patients (95 % CI: 32–48 %), followed by a spinal problem (including back symptom or complaint) for 33 % of patients (95 % CI: 28–38 %).

**Table 2:** Distribution of COAST patients’ top 20 Reasons For Encounter

RFE Group (Top 20)^a^	Number	% of patients (*n* = 3266)^b,c,d^	95 % confidence interval
[missing]	[21]		
Health maintenance/preventive care	1277	39	(32,48)
Spinal problem	1074	33	(28,38)
Neck problem	576	18	(15,21)
Shoulder problem	206	6	(5,8)
Headache	204	6	(5,8)
Hip symptom/complaint	117	4	(3,5)
Leg/thigh symptom/complaint	90	3	(2,4)
Muscle problem	83	3	(2,4)
Knee symptom/complaint	62	2	(1,3)
Arm symptom/complaint	35	1	(1,2)
Back syndrome with radiating pain	41	1	(1,12)
Sleep disturbance	40	1	(0,3)
Follow-up	34	1	(1,2)
Migraine	36	1	(1,2)
General symptom/complaint, other	30	1	(1,2)
Foot/toe symptom/complaint	33	1	(1,2)
Vertigo/Dizziness	34	1	(1,1)
Musculoskeletal symptom/complaint, other	35	1	(0,2)
Ankle problem	24	1	(0,1)
Weakness/tiredness, general	19	1	(0,2)

^a^Excludes repeat problem group managed at encounter

^b^Total number of patients for this analysis

^c^Missing values not used in percentage calculations

^d^Up to 3 RFEs could be recorded at each encounter

Table 3 shows the distribution of patient reported comorbidities by ICPC chapter. Of the 3287 patients, a total of 1049 comorbidities were recorded by 767 individuals (23 %). The majority of recorded comorbidities were coded to the musculoskeletal, circulatory and endocrine/metabolic ICPC chapters. One or more musculoskeletal comorbidity (e.g. arthritis, scoliosis) was reported for 27 % of patients (95 % CI: 22–33 %), and one or more circulatory comorbidity (including conditions such as high blood pressure and heart disease) was reported for 24 % of patients (95 % CI: 19–31 %). Comorbidities coded to the endocrine and metabolic chapter were present in 24 % (95 % CI: 20–29 %) of patients and included conditions such as diabetes, obesity and high cholesterol.

**Table 3:** COAST patient comorbidity by ICPC chapter

ICPC Chapter^a^	Number (*n* = 968)	% of patients (*n* = 767)^b,c,d^	95 % confidence interval
[missing or no comorbidity]	[2520]		
Musculoskeletal	204	27	(22,33)
Circulatory	187	24	(19,31)
Endocrine & metabolic	186	24	(20,29)
Respiratory	90	12	(9,16)
Psychological	84	11	(7,16)
Digestive	59	8	(6,10)
Neurological	52	7	(4,11)
General & unspecified	20	3	(2,4)
Skin	17	2	(1,4)
Blood	16	2	(1,4)
Male or female genital system	14	2	(1,3)
Ear	12	2	(1,3)
Pregnancy & family planning	10	1	(1,3)
Urology	9	1	(1,2)
Eye	8	1	(1,2)

^a^Excludes cases of repeat ICPC chapter

^b^Total number of patients for this analysis

^c^Missing values not used in percentage calculations

^d^Patients could have up to 3 comorbidities per encounter

Table 4 shows the lifestyle-related characteristics of COAST participants. Seventy percent (95 % CI: 67–74 %) of male and more than half (53 %; 95 % CI: 49–57 %) of female chiropractic patients were overweight or obese. Male participants in the Australian Health Survey were more likely to be underweight (1 %) or obese (28 %) than COAST patients, but were less likely to be overweight (42 %) (*χ*^2^ = 23.9, df = 3, *p* < 0.001). Female Participants in the Australian Health Survey were more likely to be obese (28 %) than COAST patients, but were similar in proportion to the underweight, normal and overweight BMI range (*χ*^2^ = 19.189, df = 3, *p* < 0.001) (Table 4).

**Table 4:** COAST patient lifestyle characteristics compared to the Australian Health Survey (AHS)

	Number	% of patients^a^	95 % confidence interval	AHS %
BMI:				
Male (*n* = 1192) ^b,c,d^				
[missing]	[33]			
Underweight (<18.5)	3	0	(0,1)	1
Normal (18.5–25)	340	29	(26,33)	29
Overweight (25–30)	551	48	(45,50)	42
Obese (>30)	265	23	(20,26)	28
Female (*n* = 1620) ^b,c,e^				
[missing]	[86]			
Underweight (<18.5)	32	2	(1,3)	2
Normal (18.5–25)	692	45	(41,50)	42
Overweight (25–30)	468	31	(28,33)	28
Obese (>30)	342	22	(19,26)	28
Cigarette smoking (*n* = 1388)^b,f^				
[missing]	[8]			
Never smoked	787	57	(54,60)	51
Used to smoke	405	29	(27,32)	31
Now smoke	188	14	(12,16)	18
Alcohol Consumption *n* = (1388)^b,g,h^				
[missing]	[59]			
Meet guidelines	491	37	(33,41)	40
Do not meet guidelines	359	27	(24,31)	20
Do not drink	479	36	(33,39)	40
Health rating (*n* = 1442)^i,j^				
[missing]	[0]			
Excellent/very good	830	58	(53,62)	55
Good	472	33	(29,36)	30
Fair/poor	140	10	(8,12)	15

^a^Missing data not used in percentage calculations

^b^Those aged 18 or more

^c^Excludes cases missing height and/or weight

^d^AHS respondents were more likely to be underweight or obese than COAST patients, but were less likely to be overweight (*χ* ^2^ = 24.9, df = 3,*p* < 0.001)

^e^AHS respondents were more likely to be obese than COAST patients (*χ* ^2^ = 19.3, df = 3,*p* = 0.001)

^f^AHS respondents were less likely to have never smoked and more likely to currently smoke (*χ* ^2^ = 22.8, df = 2,*p* < 0.001)

^g^Standard drinks in last 7 days

^h^Risky alcohol consumption was significantly lower among AHS participants (*χ* ^2^ = 48.5, df = 2,*p* < 0.001)

^i^Those aged 15 or more

^j^AHS respondents were less likely to describe their health as Excellent/Very Good or Good and were more likely to describe their health as Fair/Poor (*χ* ^2^ = 26.0, df = 2, *p* < 0.001)

More than half of COAST patients had never smoked (57 %; 95 % CI: 54–60 % ), and 14 % of COAST patients (95 % CI: 12–16 %) were current smokers. Participants of the Australian Health Survey were less likely to have never smoked (51 %) and more likely to currently smoke (18 %) (*χ*^2^ = 22.353, df = 2,*p* < 0.001).

COAST participants were more likely to engage in risky alcohol consumption with more than one quarter of participants consuming more alcohol than is recommended (27 %; 95 % CI: 24–31 %). Risky alcohol consumption was significantly lower (20 %) among participants of the Australian Health Survey (*χ*^2^ = 46.439, df = 2,*p* < 0.001).

Self-reported health was described as excellent or very good by more than half of COAST patients (58 %; 95 % CI: 53–62 %) and approximately one in 10 (10 %; 95 % CI: 8–12 %) described their health as fair or poor. Participants in the Australian Health Survey were less likely to describe their health as Excellent/Very Good (55 %) or Good (30 %) and were more likely to describe their health as Fair/Poor (15 %) (*χ*^2^ = 27.709, df = 2, *p* < 0.001).

Table 5 shows other lifestyle-related characteristics of COAST participants for which there were no comparable data available from the Australian Health Survey. Less than half of COAST patients participated in vigorous exercise at least twice per week. About two thirds participated in less vigorous exercise at least twice per week. Approximately one in five COAST patients ate one serve of vegetables or less each day, and about half ate one serve of fruit or less each day.

**Table 5:** Other lifestyle characteristics of adult (18+ years) COAST patients

	Number	% of patients^a^ (*n* = 1388)	95 % confidence interval
Vigorous exercise			
[missing]	[9]		
Never or once a week	705	51	(46,57)
2–3 times per week	381	28	(24,31)
4 or more times per week	293	21	(18,26)
Less vigorous exercise			
[missing]	[6]		
Never or once a week	411	30	(26,35)
2–3 times per week	475	34	(31,38)
4 or more times per week	496	36	(31,42)
Serves of vegetable			
[missing]	[1]		
Do not eat vegetables	16	1	(1,2)
1 serve or less	275	20	(17,23)
2–3 serves	746	54	(50,58)
4 or more serves	350	25	(21,30)
Serves of fruit			
[missing]	[2]		
Do not eat fruit	79	6	(4,8)
1 serve or less	578	42	(39,45)
2–3 serves	649	47	(44,50)
4 or more serves	80	6	(5,7)

^a^Missing data not used in percentage calculations

## Discussion

In this study we have described who consults chiropractors, the reasons people consult chiropractors, and the health profile and lifestyle factors of a sample of chiropractic patients in Victoria, Australia. The typical chiropractic patient is female, between the ages of 25 to 64, and is wealthier and healthier than the general population. Patients mostly attend chiropractors for care related to specific musculoskeletal problems, followed by care related to maintaining good health. While chiropractic patients tend to be healthier than the general population overall, they still report concerning levels of obesity, smoking, alcohol consumption and other lifestyle related problems.

Consistent with the few published studies describing reasons for attending chiropractors, COAST patients mainly sought care for musculoskeletal complaints [5, 6]. However, just over a third of patients were consulting for preventative care without any specific symptom or complaint. This is despite the lack of evidence supporting chiropractic treatment for health maintenance or preventive care, particularly when it comes to providing spine-only care for general health improvement [23]. Understanding the reasons for seeking such preventative care requires qualitative research involving patients and chiropractors. This research would help to further understand the chiropractic profession’s role in the healthcare system.

Using COAST results as an indicator, there are many opportunities for chiropractors to reinforce health promoting messages with their patients due to the commonly coinciding problems of obesity, smoking, excessive alcohol consumption, inactivity and poor diet. Despite COAST participants being healthier than the general population, a higher proportion exhibited unsafe drinking levels. In Australia, alcohol consumption is a major cause of preventable morbidity and mortality [24]. Alcohol contributes to more than 3000 deaths and 100,000 hospitalisations each year and the rates of acute and chronic harms related to alcohol are increasing [25]. Alcohol consumption and associated risk factor studies have shown links between increased alcohol consumption and both lower and higher socioeconomic status (SES) [26–29]. Further investigation of alcohol consumption amongst COAST participants is outside the scope of this study. However, with more than a quarter of COAST participants consuming more alcohol than is recommended, there is a clear public health opportunity for chiropractors to encourage reduced alcohol consumption by their patients.

Australian NHMRC guidelines recommend two or more serves of fruit and five or more serves of vegetables a day [30]. Recent guidelines also advise minimising sedentary behaviour, suggesting 150–300 minutes of moderate intensity physical activity each week [31]. While COAST data were not able to be compared directly to NHS data for these factors, our results demonstrate that a considerable proportion of COAST participants were failing to meet these guidelines. These results indicate that chiropractors may also have the opportunity to provide health promotion advice to patients about these lifestyle behaviours.

There are multiple possible explanations for COAST participants having more positive health and lifestyle characteristics than the Australian population. Arguably the most likely explanation is that COAST patient participants came from areas of higher SES, as indicated by higher SEIFA deciles. Generally, people in areas of higher SEIFA have higher incomes, are more educated, are more likely to meet physical activity guidelines, have better health than the general population and are more likely to use healthcare services [16, 32–34]. While other studies describing chiropractic practice have incorporated a SES measure into their analyses [4, 35], they typically use annual income. A strength of our analysis in using SEIFA is that this takes into account additional SES measures such as education and employment levels [16]. Another possible explanation of COAST participants having more positive health and lifestyle characteristics than the Australian population may be that patients making their own health choices and committing time and finances to seeking health care place different emphasis on health. They therefore have a different perception of their own role in maintaining their health compared to those who do not.

While COAST patient participants were more likely to come from less disadvantaged areas (higher SEIFA decile), and were more likely to work, less than half used private health insurance at their chiropractic encounter. This lower than expected uptake of health insurance for chiropractic services may be related to how many visits are covered by the insurance company. For example, at the time of COAST data collection one company, Medibank private, had a limit of AUD$300 a year for chiropractic, osteopathy, acupuncture, naturopathy and remedial massage combined [36]. Regular visits to one or more of these providers may result in health insurance not being used at later treatments and patients needing to pay for their treatment directly.

### Strengths and limitations

COAST is the first Australian study to use a rigorous, established primary care observational study methodology to describe who uses chiropractic services and why they use them. Although the reliability and validity of the chiropractic encounter forms was not evaluated in this particular study, the forms were based on encounter forms used in a long-standing study of Australian general medical practice clinical activity with an established reliable and valid data collection process [37]. This study adds to the knowledge base by comparing chiropractic patients to the general population, and identifies SES as a factor to take into account when analysing chiropractic patient demographics. A limitation of this study was the potential confounding influence of SES on the analysis. While incorporating weighting adjustment to limit the effect of SEIFA score on the comparative analysis, the differences shown between COAST patient participants and the general population may still be due to them having a higher SES.

As reported in the original results paper, the response rate for this study was 33 % of the eligible chiropractors approached [11]. Although this was lower than desired, we demonstrated that the participating chiropractors appear to be generally representative of the Australian chiropractic population [11]. However, there are currently no representative Australian chiropractic patient data available to which we can compare the patient characteristics of our study, so we are unable to confidently determine if the patients in the current study were also representative of the Australian chiropractic patients. We also do not have any information about chiropractors who were approached but did not choose to participate in the study, which could have been another way of determining possible selection bias in this study. Further practice-based studies in the Australian chiropractic profession are needed to confirm or refute our results.

While the overall majority of RFEs were for musculoskeletal conditions, the most common single RFE reported was for health maintenance, suggesting that the patient is undertaking chiropractic treatment for a perceived preventative health benefit. However, a limitation of measuring this variable is that because RFE was a self-reported category the consultation could have been sought for maintenance of an ongoing musculoskeletal condition such as neck or back pain, or the patient may have had no current health complaint and was seeking care for general health maintenance (patient defined). The nature of the data collection meant that separation into either category was not possible.

This study had the potential for selection bias with chiropractors collecting data from their own patients. While chiropractors were instructed to collect information from consecutive patient encounters, we do not know whether some patients were selectively excluded by the participating chiropractors. In addition, we have no way to tell if certain encounter fields, e.g. comorbidity, were left blank because the patient had no coexisting health condition, because the patient refused to have comorbidity recorded, or if the chiropractor did not ask this question at that encounter.

This study includes all patients presenting to chiropractors and the reason they present, not just the reasons new patients present to chiropractors. Hence, patients attending more often during the study period were more likely to be sampled. If the study was only of new patients, it is likely that a large majority of patients would be presenting with a specific health condition, rather than for maintenance. Therefore, a similar study sampling only new patients would be likely to result in a higher proportion presenting as new patients for care with a specific health condition.

### Implications for chiropractic policy, practice and research

For chiropractic practice, chiropractors can compare their own practice to that of their peers and explore the reasons for similarities and differences. This study provides justification for chiropractors to provide evidence-based health promotion to their patients, particularly around diet, smoking and alcohol consumption. For chiropractic healthcare policy, COAST findings can direct provision of services to areas of greatest need. For example, it is indicated that many chiropractic patients would benefit from health promotion activities, however, not many chiropractors are actually using evidence-based health promotion interventions to address these issues, and chiropractors may be ill-equipped to implement these activities in their practice [23]. Education and support of these health promotion activities are required to maximise the opportunity to improve the health of people who seek chiropractic care. Further, improved communication between chiropractors and other primary healthcare providers who may co-manage these patients would help to improve their health promotion efforts. Finally, considering the large number of patients who consult chiropractors for wellness/health maintenance with no apparent symptoms, robust, large scale, clinical studies are needed to determine the effects of chiropractic treatment for this population.

## Conclusions

COAST patient participants were healthier than the general population, but drinking alcohol excessively was common. A better health profile than the general Australian population on most health indicators was most likely related to chiropractic patients living in areas of higher SES. The poor lifestyle habits in meeting alcohol, fruit and vegetable guidelines highlight important areas where chiropractors have the opportunity to make a difference to the health of their patients through evidence-based health promotion and education of healthy lifestyle behaviours.

## Abbreviations

ABS, Australian Bureau of Statistics; AUD, Australian Dollar; BMI, Body Mass Index; CI, Confidence Interval; COAST, Chiropractic Observational and Analysis STudy; ICPC-2 PLUS, International Classification of Primary Care, Version 2 through the Australian ‘PLUS’ general practice terminology; ICPC-2, International Classification of Primary Care, Version 2; NHMRC, National Health and Medical Research Council; NHMS, National Health Measures Survey; NHS, National Health Survey; NNPAS, National Nutritional and Physical Activity Survey; RFE, Reason For Encounter; RSAD, Relative Socio-economic Advantage and Disadvantage; SEIFA, Socio-economic Indices for Areas; SES, Socioeconomic Status.
